# Considerations on surgical strategies and associated risk profiles for endoscopic tumor biopsies within the third ventricle and periaqueductal region

**DOI:** 10.1007/s00381-023-06122-9

**Published:** 2023-09-08

**Authors:** Fritz Teping, Joachim Oertel

**Affiliations:** Department of Neurosurgery, Faculty of Medicine, Saarland University, Kirrbergerstraße, Building 90.5, D-66421 Homburg, Germany

**Keywords:** Pediatric, Neuroendoscopy, Ventricular surgery, Endoscopic tumor biopsy

## Abstract

**Introduction:**

Neuroendoscopic techniques have proven to be a successful and minimally-invasive technique for tumor biopsies within the third ventricle in pediatric patients. However, a comprehensive assessment of associated surgical strategies, techniques, and morbidity is essential to optimize patient outcomes.

**Methods:**

This retrospective study analyzed full endoscopic tumor biopsies in pediatric patients with tumors in the third ventricle and periaqueductal region. Data from 1995 to 2022 were collected from medical records, imaging, and intraoperative video documentation.

**Results:**

In this study, 16 shear endoscopic tumor biopsies were performed using the transventricular transforaminal approach. Tumors were located in the anterior or mid part of the third ventricle (50%) or in the periaqueductal and pineal recess region (50%). Preoperative hydrocephalus was seen in 81.25%. Tumor biopsies were harvested successfully in all cases. Simultaneous ETV was performed in 12 (75%) cases and additional septostomy in 3 (18.75%). Significant intraoperative bleeding occurred in 3 cases (18.75%). All bleeding situations could be successfully managed with continuous irrigation. Histopathology revealed astrocytoma as the predominant diagnosis (75%). No new neurologic deficits were observed, except for one case of transient oculomotor nerve paralysis after ETV. Hydrocephalus persisted in 18.6% of all cases with the need of urgent ventriculoperitoneal shunting in two patients.

**Conclusion:**

In conclusion, neuroendoscopy emerges as an effective technique for tumor biopsies within the third ventricle in pediatric patients, offering the added advantage of simultaneous treatment of obstructive hydrocephalus. However, it is essential to acknowledge the specific intra- and postoperative risks associated with various surgical strategies. The safe management and achievement of favorable clinical results demand extensive experience and expertise.

## Introduction

Pediatric brain tumors situated within the third ventricle and periaqueductal region present intricate challenges in neurosurgical management due to their delicate proximity to vital neural structures. To address these intricacies, endoscopic techniques have emerged as widely implemented modalities, offering enhanced visualization and minimally invasive access even to deep-seated lesions [1–6]. However, alongside the benefits, it is essential to recognize and address the associated risks and morbidities inherent in endoscopic tumor biopsies in this vulnerable patient population [7]. By analyzing and discussing the presented case series, we strive to provide valuable insights to enhance risk assessment, guide clinical decision-making, and optimize patient safety and outcomes in this challenging neurosurgical context.

## Methods

### Study design and patient selection

This retrospective study focuses on endoscopic intraventricular procedures performed on pediatric patients who underwent tumor biopsies within the third ventricle and periaqueductal region. The study spans a period from 1995 to 2022, including surgeries of the senior author conducted at four medical centers: University of Greifswald, Nordstadt Hospital Hannover, University Medical Center Mainz, and Saarland University Medical Center. Patient selection criteria encompassed individuals below 18 years of age at the time of surgery who underwent an endoscopic biopsy for tumors located within the specified regions as part of their oncologic therapy concept.

### Data management and statistical analysis

To collect comprehensive data, a meticulous review of medical records, radiographic imaging, and intraoperative video documentation was conducted. The gathered data were organized in a retrospective database, and SPSS (IBM, Armonk, USA) was utilized for data management and analysis.

Given the limited number of cases, the statistical analysis primarily relied on descriptive statistics. Additionally, to provide a comprehensive understanding of each case, individual case presentations were thoughtfully employed, considering the unique and rare nature of these cases.

The research adhered to the guidelines outlined by the Saarland Ethics Committee, and patient data were meticulously anonymized to uphold confidentiality and comply with data protection regulations.

### Surgical technique

Surgeries were performed utilizing the Karl Storz Hopkins rod lens system (Karl Storz Endoskopie, Tübingen, Germany) and corresponding endoscopic instruments. In cases of transaqueductal inspections, a flexible neuro-fiberscope was used (Karl Storz Endoskopie, Tübingen, Germany). A detailed description of the surgical technique has been previously published [8]. To ensure consistency and minimize procedural variations, all surgeries were exclusively performed by the senior author (J.O).

## Results

A total of 16 fully endoscopic tumor biopsies were performed in pediatric patients, employing the transventricular transforaminal approach for all surgeries. The study population consisted of 9 female and 7 male patients, with a mean age at surgery of 10.8 years (± 5.3 years). Among the cases, 8 tumors were predominantly located in the third ventricle, while the remaining 8 tumors were situated in the periaqueductal region. Preoperative CSF pathway obstruction with hydrocephalus was present in all but 3 cases, and common preoperative symptoms included headaches (62.5%), nausea (43.75%), and vomiting (31.25%) as typical signs of hydrocephalus. The general information on the study population is provided in Table 1.

**Table 1:** Characteristics of the study population

Characteristics		Procedures (*n* = 16)
Male: female		7:9
Age at surgery		10.8 years (± 5.3 years)
Pathology	Astrocytoma	12 (75%)
	Germinoma	2 (12.5%)
	Neurinoma	1 (6.25%)
	Unspecified gliosis	1 (6.25%)
Tumor location	Anterior or mid part of the third ventricle	8 (50%)
	Periaqueductal region	8 (50%)
Preoperative hydrocephalus		13 (81.25%)
Surgery	Completed tumor biopsy	16 (100%)
	Additional ETV	12 (75%)
	Additional septostomy	3 (18.75%)
	Significant intraoperative bleeding	3 (18.75%)
	Surgical time	35–135 min
Additional CSF pathway restoration	Postoperative clinical improvement	13 (81.25%)
	Persistant hydrocephalus with need for Shunting	3 (18.75%)
	Wound healing disorder with CSF fistula	1 (6.25%)
	Transient oculomotor nerve palsy	1 (6.25%)
	Surgical mortality	0 (0%)

In all cases, the fully endoscopic transventricular transforaminal tumor biopsy was conducted following the previously published technique [8]. Neuronavigation was implemented in all cases for planning of the ideal trajectory and entry point. Additional ETV was performed in 12 cases (75%), while 3 cases (18.75%) underwent additional septostomy. A stent-supported aqueductoplasty was performed in one case (6.25%). All surgeries were successfully completed without the need for premature termination, with surgical times ranging from 35 to 135 min. Histopathology results indicated astrocytoma as the most common diagnosis, accounting for 75% of the cases.

During the intraoperative phase, significant bleeding occurred in 3 cases (18.75%). Timely and effective management through continuous irrigation, lasting up to 40 min, allowed successful control of the bleeding. Postoperatively, hydrocephalus-associated symptoms improved in all but 3 cases (18.75%). However, 2 cases (12.5%) exhibited progressive hydrocephalus with associated clinical deterioration, necessitating urgent secondary shunt placement, while the third case required shunt placement during the further treatment period. Additionally, one child developed a CSF fistula, necessitating surgical wound revision. Furthermore, there was one case of transient paralysis of the oculomotor nerve following additional ETV. Other than these specific instances, no new neurologic deficits were observed postoperatively. The most common postoperative clinical finding was fluctuating headaches, mainly associated with the presence of the tumor itself.

### Case demonstration 1

**Fig. 1:**
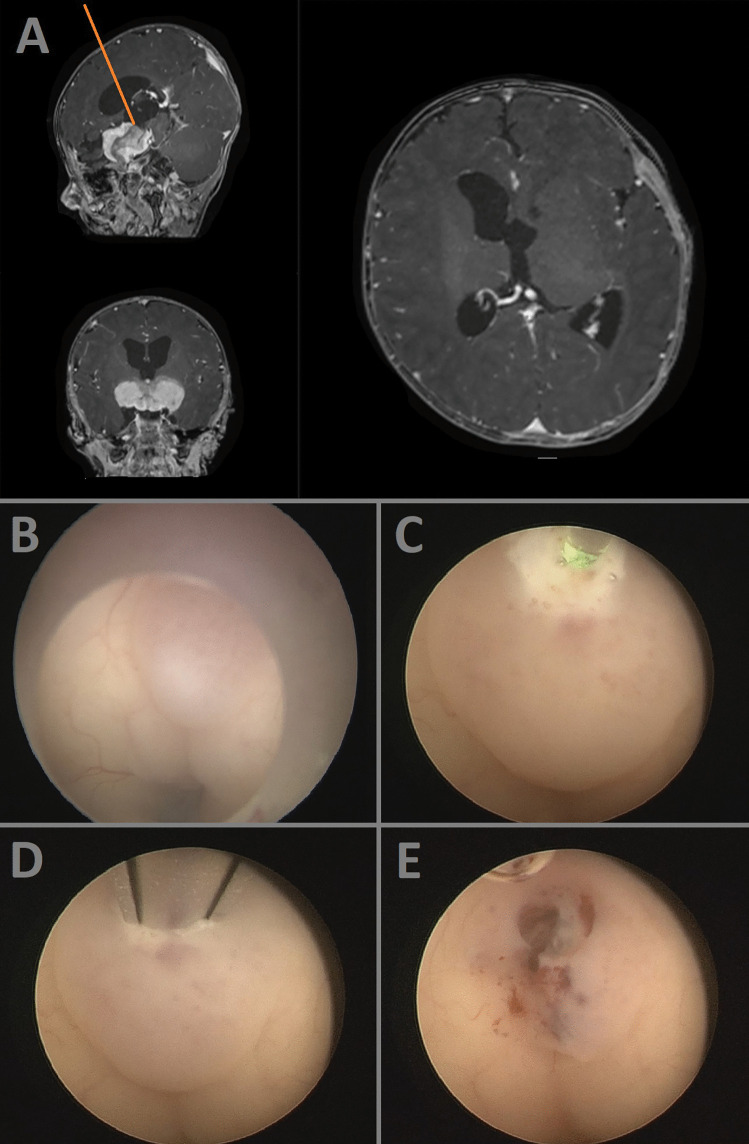
Pre- and intraoperative illustrations of the case demonstration 1. The surgical trajectory is marked with orange lines

A 4-month-old male infant was admitted to the pediatrics department due to persistent nausea and vomiting. Neuroimaging via MRI revealed an extensive tumor lesion in the suprasellar region, extending into the third ventricle (Fig. 1A). To establish a histologic diagnosis, a transventricular biopsy was scheduled. The surgical strategy involved performing the biopsy through the lamina terminalis, precisely where the tumor was reaching the ependyma (Fig. 1B). To prevent significant bleeding during the procedure, the biopsy location was coagulated using a laser (Fig. 1C). Subsequently, the biopsy was taken with the sedan probe (Fig. 1D). Post-biopsy inspection showed no residual bleeding, and sufficient tumor material was successfully harvested (Fig. 1E). Histopathologic analysis of the biopsy revealed a diagnosis of pilomyxoid astrocytoma. Following a thorough evaluation of therapeutic opportunities with the family, a treatment plan involving tumor debulking and ventriculoperitoneal shunt placement was scheduled for the further clinical course.

### Case demonstration 2

**Fig. 2:**
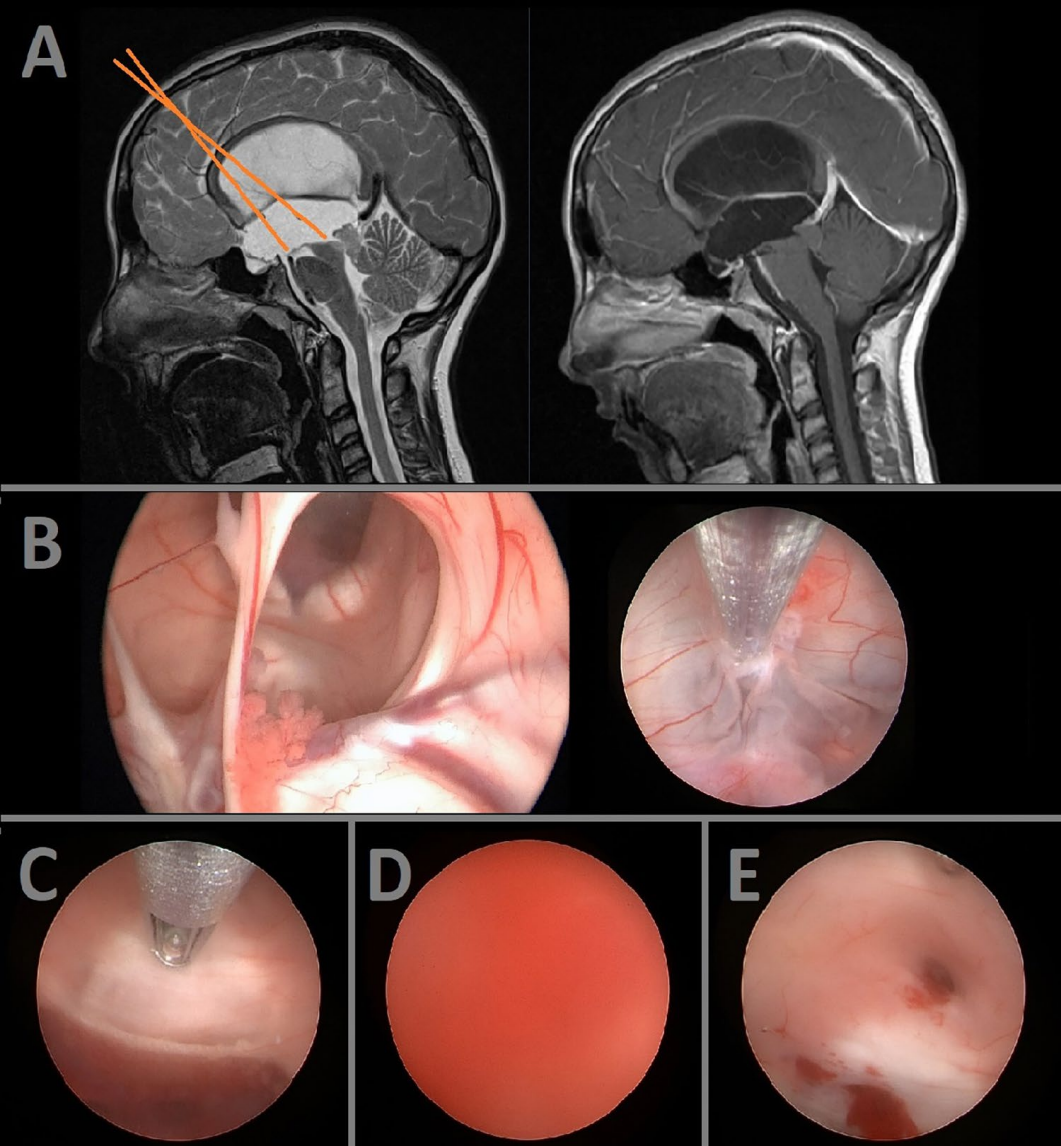
Pre- and intraoperative illustrations of the case demonstration 2. The surgical trajectory is marked with orange lines

A 15-year-old female patient was admitted to the pediatrics department with complaints of nausea and vomiting. Neuroimaging via MRI revealed the presence of a significant periaqueductal lesion causing obstructive hydrocephalus with consecutive aqueductal stenosis (Fig. 2A). To obtain a histologic diagnosis, a transventricular biopsy was scheduled. Upon inspection of the ventricles, the extent of hydrocephalus was already apparent, characterized by the degenerated interventricular septum and dilation of the third ventricular floor (Fig. 2B). To address the hydrocephalus, an ETV was performed to restore the CSF flow (Fig. 2B). Subsequent inspection of the aqueduct using angled optics allowed clear identification of the tumor. The biopsy was conducted using the sedan probe (Fig. 2C). During the biopsy, a significant episode of bleeding occurred resulting in intraoperative redout (Fig. 2D). However, prompt intervention through patient and constant irrigation over approximately 20 min enabled the reestablishment of sufficient visualization to complete the biopsy procedure (Fig. 2E). The patient’s postoperative course remained uneventful, with successful CSF restoration achieved through ETV. Histopathologic analysis of the biopsy sample revealed a diagnosis of pilocytic astrocytoma. As a result of effective CSF restoration via ETV, no further CSF diversion procedures were required.

### Case demonstration 3

**Fig. 3:**
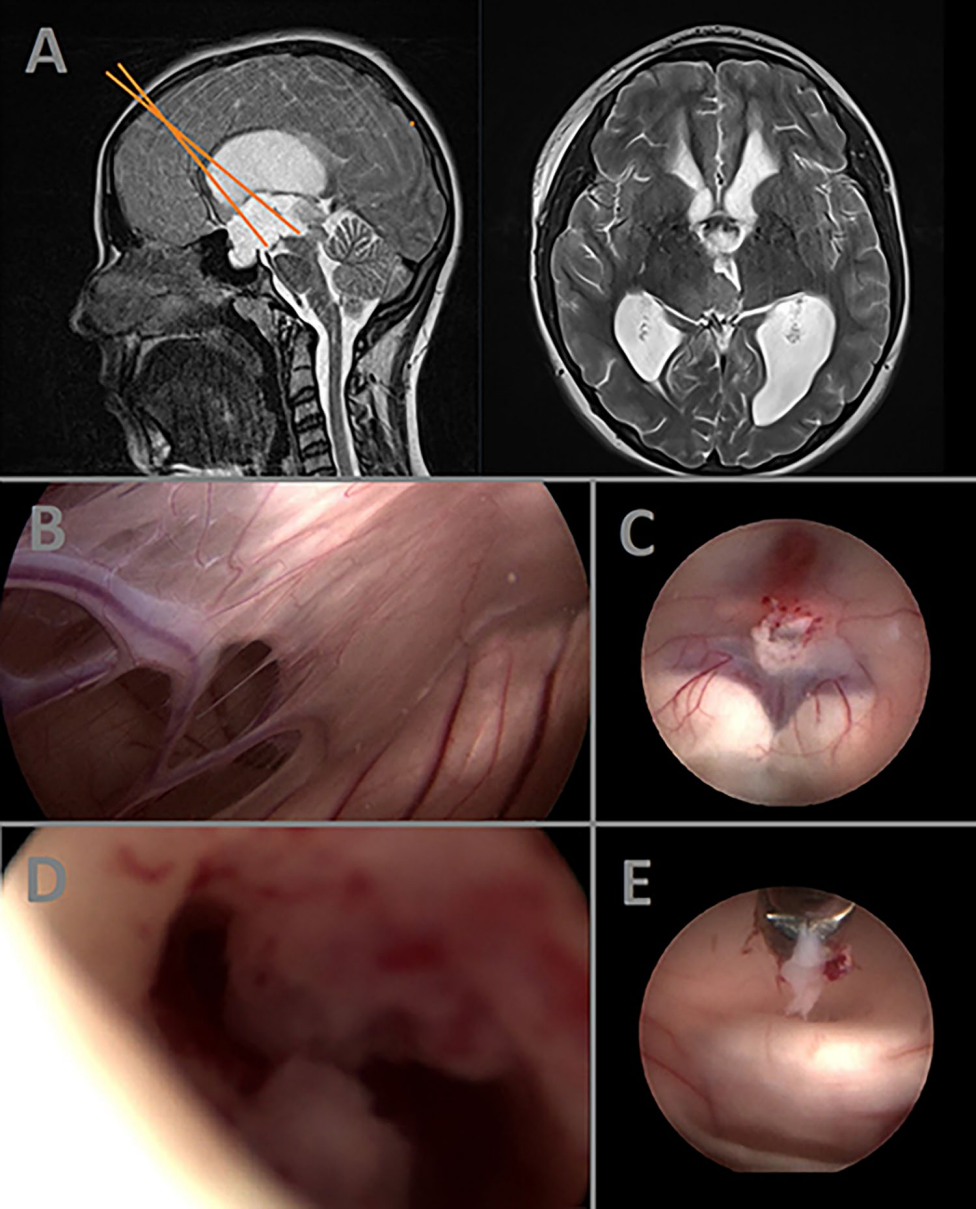
Pre- and intraoperative illustrations of the case demonstration 3. The surgical trajectory is marked with orange lines

A 14-year-old female patient presented with diffuse headaches and concentration deficits, prompting admission to the pediatrics department. Neuroimaging via MRI revealed the presence of an intra-aqueductal lesion causing hydrocephalus (Fig. 3A). To obtain a histologic diagnosis, a transaqueductal biopsy was scheduled. Intraoperatively, the degenerated septum interventriculare was found resulting from chronic hydrocephalus (Fig. 3B), despite the relatively mild clinical symptoms. To address the hydrocephalus and restore the cerebrospinal fluid (CSF) pathway, an endoscopic third ventriculostomy (ETV) was performed (Fig. 3C). With the aid of angled optics, close-up high visualization of the aqueduct allowed for precise identification of the tumor (Fig. 3D). Subsequently, a biopsy was performed using grasping forceps (Fig. 3E). The patient’s postoperative course remained uneventful, and histopathologic analysis of the biopsy sample revealed a diagnosis of pilomyxoid astrocytoma. However, during the further clinical course, the tumor exhibited significant extension into the fourth ventricle, necessitating tumor resection.

## Discussion

The presented findings demonstrate the overall successful implementation of the fully endoscopic transventricular transforaminal approach in all cases, enabling direct access to tumors within the entire third ventricle including the aqueduct. Depending on the entry point, this approach has been proven effective in pediatric neurosurgery, offering precise visualization and maneuverability while minimizing the need for extensive craniotomies [8, 9]. However, preoperative planning of the ideal trajectory remains of utmost importance to maintain patient’s safety while achieving surgical success.

### CSF pathway restoration and shunt dependency

For (peri)-aqueductal lesions with occlusive hydrocephalus a simultaneous ETV should be considered for CSF pathway restoration. The benefits and risk profile of ETVs has been widely covered within literature [10–15]. In recent years, the traditional perforation method with bipolar coagulation has been partly replaced by modern laser techniques such as neodymium–yttrium/aluminum/garnet (Nd–YAG) lasers [16]. It has to be kept in mind that in cases of suprasellar tumors with deranged anatomy a potential thermal irritation of neural structures, such as the oculomotor nerve, may lead to transient palsies postoperatively, as shown in this series. However, simultaneous ETV serves as an effective option to restore CSF pathway in tumorous aqueductal stenosis and may avoid shunting procedures.

Aqueductal stenting should be considered in specific cases were stent placements can be performed without harming the surrounding midbrain. The authors highly recommend using assisting techniques to ensure safe manipulation and optimal placement of the stent, such as the intra-catheter ShuntScope [17–19].

However, despite all surgical effort, 18.8% of the presented patients needed secondary shunt implantations. This finding is mainly attributable to the tumorous cause of the aqueductal stenosis leading to higher ETV failure rates compared to other benign causes [20]. The fact that two of those cases developed rapid clinical deterioration with subsequent emergency surgery emphasizes the importance of a meticulous postoperative surveillance.

### Intraoperative bleeding

Tumor biopsies come along with an increased risk of intraoperative bleeding. As common for intraventricular endoscopy, bleeding may have significant impact on the further course of surgery. Most feared complication is the so-called persistent redout with complete loss of visual orientation. In the presented study, significant bleeding after tumor biopsy occurred in 18.8% of the cases. However, most effective control can be gained by maintaining patience and constant irrigation by the assistant [21]. In some cases, irrigation periods can last more than 30 min. If a distinct bleeding vessel within the tumor mass can be identified, local bipolar or laser coagulation can also lead to an effective bleeding control.

### Anatomical orientation, neuronavigation, and image guidance

For the planning of the ideal trajectory and according burr hole location, implementation of neuronavigation should be considered the gold standard. Especially when it comes to combined procedures of tumor biopsies in the periaqueductal region and ETV a sufficient range of mobility with the endoscope is of utmost importance. In cases of narrow configuration of the ventricular system due to the absence of hydrocephalus, frameless navigation has shown to reduce surgical morbidity [22]. For special indications of tumors not reaching the ependymal surface, Di Somma et al. proposed an ultrasound-guided technique as an additional guidance tool [23]. However, intraventricular anatomy can be significantly distorted due to chronic hydrocephalus itself or previous bleeding or infection. In such cases meticulous and patient ventriculoscopy should be performed prior to any intervention to achieve sufficient orientation for any further steps.

### Training and skill development

The significance of training and skill development in endoscopic techniques cannot be underestimated. Complication rates and failures are mainly found during the early experience of the surgeon [13]. In particular, managing complications such as intraoperative bleeding and implementing effective control techniques require expertise gained through experience. Many courses and hands-on workshops are offered by high-level centers and international foundations to enable younger neurosurgeons a safer and more effective entry into neuroendoscopy. Furthermore, with ongoing development of technology and virtual reality, even more effective training and planning opportunities can be offered [24, 25]. Continuous education, simulated training, and mentorship, as well as a continued commitment to skill development ensure patient’s safety and optimal outcomes, fostering the dissemination of best practices in the field.

### Multidisciplinary collaboration

Another vital aspect of risk management for pediatric patients after intraventricular tumor biopsy is the multidisciplinary approach. Although not directly associated with surgical morbidity, an informed and close cooperation between pediatricians and neurosurgeons is essential for the final outcome. Early identification of complications or clinical deterioration within the early postoperative course is the key to an effective management. A proactive approach to postoperative surveillance enables the timely initiation of relevant diagnostics, ensuring the prompt detection of any signs warranting surgical re-intervention, if necessary.

## Conclusion

Endoscopic tumor biopsies within the third ventricle and periaqueductal region in pediatric patients offer a feasible and successful approach with precise visualization and minimized invasiveness. Occlusive hydrocephalus can be treated simultaneously and intraoperative complications can be addressed effectively. However, individual neuroendoscopic experience and effective interdisciplinary management are crucial for the final clinical outcome.

## Abbreviations

CSF: Cerebrospinal fluid
ETV: Endoscopic third ventriculostomy
